# Long-term outcomes of trabeculectomy versus canaloplasty in open-angle glaucoma – an 11-year follow-up of the TVC study cohort

**DOI:** 10.1186/s12886-025-04183-9

**Published:** 2025-06-16

**Authors:** Raoul Verma-Fuehring, Juliane Matlach, Thomas Klink, Jost Hillenkamp, Franz Grehn

**Affiliations:** 1 Department of Ophthalmology, University Hospital Würzburg, Würzburg, Germany; 2 Department of Ophthalmology, University Hospital Leipzig, Leipzig, Germany; 3 Eye Hospital Herzog Carl Theodor, Munich, Germany

**Keywords:** Glaucoma, Trabeculectomy, Canaloplasty, Intraocular pressure, TVC Study

## Abstract

**Purpose:**

To assess the long-term outcomes of the Trabeculectomy versus Canaloplasty (TVC) study cohort after 11 years. The TVC study was a prospective, single-center, randomized clinical trial comparing the 24-month surgical outcomes of trabeculectomy and canaloplasty in patients with open-angle glaucoma.

**Methods:**

This prospective follow-up study included only patients from the original 2015 TVC cohort. Primary endpoints were complete (without glaucoma medication) and qualified success (with or without glaucoma medication), defined as intraocular pressure (IOP) ≤ 18 mmHg (Definition 1) or IOP ≤ 21 mmHg with ≥ 20% reduction from baseline (Definition 2). Secondary endpoints included changes in IOP, medication use, complications, and revision surgeries.

**Results:**

Mean follow-up was 11.9 ± 1.1 years for trabeculectomy (TE) and 11.0 ± 2.0 years for canaloplasty (CP) (15 TE and 13 CP patients). Complete success rates for TE vs. CP were 53.3% vs. 15.4% (Definition 1, p = 0.06) and 46.7% vs. 15.4% (Definition 2, *p* = 0.22). Qualified success was achieved by 73.3% vs. 69.2% (Definition 1, *p* = 1.0) and 66.7% vs. 76.9% (Definition 2, *p* = 0.69). Median IOP was 10.0 (6.0–12.0) mmHg for TE and 14.0 (11.5–17.75) mmHg for CP (*p* < 0.01). Mean number of compounds was 1.0 ± 1.4 in TE and 1.9 ± 1.5 in CP (*p* = 0.17). Revision surgeries were needed in 26.7% (TE) vs. 23.1% (CP). The only long-term complication, hypotony maculopathy, occurred in two TE patients (15.4%).

**Conclusion:**

After 11 years, trabeculectomy still demonstrated greater IOP reduction and higher complete success rates but was associated with a higher complication rate. In contrast, canaloplasty offers a safe alternative when slightly higher IOPs and moderate medication use are acceptable.

**Supplementary Information:**

The online version contains supplementary material available at 10.1186/s12886-025-04183-9.

## Main text

### Introduction

Surgical intervention remains a fundamental aspect of glaucoma management, with trabeculectomy and canaloplasty as two widely used techniques. While trabeculectomy is a primary fistulation procedure, canaloplasty aims to enhance conventional trabecular outflow [1].

The 2015 Trabeculectomy versus Canaloplasty (TVC) study demonstrated that both surgical procedures effectively reduced intraocular pressure (IOP) in patients with open-angle glaucoma over two-years [2]. Trabeculectomy achieves greater IOP reduction with fewer medications, but involves higher complication rates and more intensive postoperative care than canaloplasty [3, 4].

Randomized trials, such as the TVC study, are crucial for guiding clinical decision-making in glaucoma management. These studies directly compare trabeculectomy, a widely established standard of care, with less invasive techniques or therapy regimens, providing essential insights into their relative strengths and limitations [5, 6].

Despite valuable insights provided, many trials focus on short- to medium-term outcomes, leaving critical gaps in understanding the durability of therapeutic effects. The long-term trajectory of IOP control, the sustained need for medications, and the cumulative burden of complications are key parameters that remain largely unexplored.

This study aims to evaluate the long-term efficacy and safety of trabeculectomy and canaloplasty in the original TVC cohort, contributing to personalized patient care and guide surgical decision-making in glaucoma management.

## Methods

### Ethics approval

This study was conducted in accordance with the tenets of the Declaration of Helsinki and received approval from the institutional review board of the University of Würzburg (#3/23-sc). Before inclusion in the study, all participants provided informed consent.

### Study design

This is a long-term prospective follow-up study of the cohort originally enrolled in the 2015 Trabeculectomy versus Canaloplasty (TVC) study. The TVC study compared the efficacy and safety of trabeculectomy and canaloplasty in patients with open-angle glaucoma. The objective of this study was to assess the extended outcomes of these procedures and compare them to the 12-month outcomes of the original study.

### Study population and patient recruitment

Original TVC study patients were invited for a follow-up examination at the Department of Ophthalmology, University Hospital Würzburg, via an invitation letter with study details and a consent form. Travel costs were reimbursed. Standardized information on IOP, visual acuity, glaucoma medications, and surgical interventions or complications was obtained. For non-attendees, data was collected from local ophthalmologists. Only consenting patients with complete and consistent data were included, while those with incomplete data or method inconsistencies were excluded. The recruitment period ran from March 2023 to October 2024.

### Data collection

Patients undergoing follow-up at our Department of Ophthalmology underwent comprehensive ophthalmic examinations. A single examiner (RVF) and a study nurse conducted the examinations.

The examination documented current IOP-lowering compounds and additional glaucoma surgeries since the original study. Best-corrected visual acuity (BCVA) was assessed using standard protocols.

The anterior segment was examined by slit lamp, including the state of filtering blebs and the anterior chamber angle. A dilated fundus examination was performed.

IOP was measured on two separate occasions using Goldmann applanation tonometry with a one-hour interval between each measurement. One value was obtained in miosis, and the other after the instillation of tropicamide eye drops. As in the original TVC study, no washout of glaucoma medication was performed at baseline.

Spectral-Domain Optical coherence tomography (SD-OCT) was performed using Heidelberg Spectralis OCT (Heidelberg Engineering, Heidelberg, Germany). The G-value of the retinal nerve fiber layer (RNFL) scan was used as a descriptive value in the results. Only scans with a Q value of > 20 were included. Visual field testing was conducted using a static perimeter with the 30–2 program (Octopus 900, Haag-Streit, Switzerland).

For patients whose data were collected from external ophthalmologists, standardized forms were provided to ensure consistent documentation of key parameters, including IOP, visual acuity, OCT, visual field, and relevant surgical or treatment history.

### Outcome parameters

Success rate was defined as the primary endpoint. Overall criteria for success were established, requiring an IOP of ≥ 5 mmHg, no additional glaucoma surgery, and the preservation of light perception. After fulfilling these overall criteria, success was further defined in two ways:

- IOP ≤ 18 mmHg (Definition 1)
- IOP ≤ 21 mmHg AND a reduction of at least 20% from baseline (Definition 2)

Success was categorized as complete when achieved without glaucoma medication, and as qualified when achieved regardless of glaucoma medication use.

Secondary outcomes included the absolute change in IOP from baseline to follow-up, changes in the number of glaucoma medications, visual field mean defect (MD), and best-corrected visual acuity (BCVA).

Furthermore, the incidence of long-term complications and the need for revision surgery were assessed.

### Statistical analysis

The Department of Statistics at the University of Würzburg calculated the sample size to ensure 80% power at a 0.05 significance level, requiring 50% of the original TVC 2015 cohort for follow-up. Statistical analyses compared long-term outcomes between the trabeculectomy (TE) and canaloplasty (CP) groups using OriginPro (Version 2023b, OriginLab, Massachusetts, USA). Continuous variables were reported as mean ± standard deviation or median (IQR). The Mann–Whitney U test was used for cross-sectional analysis, while the Wilcoxon signed-rank test handled longitudinal comparisons. Fisher’s Exact test identified differences in dichotomous variables. Kaplan–Meier analysis assessed cumulative success probability, with the log-rank test comparing survival distributions. A *p*-value ≤ 0.05 indicated statistical significance for all analyses.

## Results

**Fig. 1:**
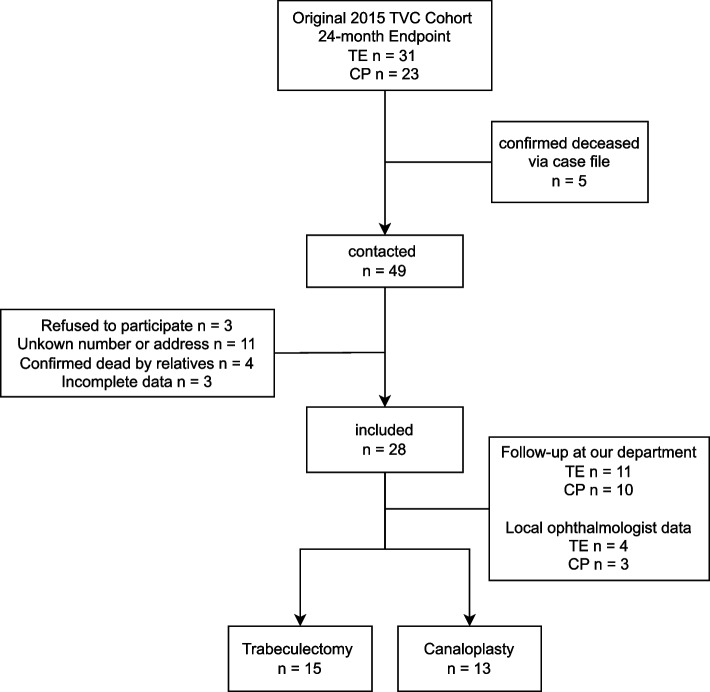
Flowchart of patient Enrolment. Enrolment process for our study cohort. Starting with the original 2015 TVC cohort (TE *n* = 31, CP *n* = 23), patients were excluded due to confirmed death via case files, refusal to participate, unknown address, or confirmation of death by relatives. Of 49 patients contacted, 28 were included in the analysis. Follow-up data were obtained either through visits to our department (TE *n* = 10, CP *n* = 11) or from local ophthalmologists (TE *n* = 3, CP *n* = 4). The final cohort at long-term follow-up consisted of 13 patients in CP and 15 patients in TE. Abbreviations: TE = Trabeculectomy, CP = Canaloplasty

Figure 1 provides an overview of the patient enrolment process. The final cohort at long-term follow-up consisted of 13 patients in CP and 15 patients in TE. Baseline demographics and parameters are shown in Table 1. The mean time for follow-up was 11.9 ± 1.1 years in TE and 11.0 ± 2.0 years in CP, respectively. Overall success was achieved in 73.3% in TE and 76.9% in CP.

**Table 1:** Demographic baseline parameters for both groups

Parameter	Trabeculectomy (*n* = 15)	Canaloplasty (*n* = 13)	*P*-value
Age [years]	62.7 ± 9.0	64.5 ± 11.2	0.65
male/female [%/%]	33.3/66.7	53.9/46.2	0.24
Median Baseline IOP [mmHg]	20.0 (16.0–21.0)	23.0 (21.5–28.0)	0.22
BCVA [logMAR]	0.1 ± 0.2	0.2 ± 0.3	0.44
Compounds [n]	3.1 ± 0.8	2.2 ± 1.5	0.07
Glaucoma Type [%]			
POAG	46.7 (7/15)	46.2 (6/13)	0.99
PXG	47.7 (7/15)	46.2 (6/13)	0.99
PG	6.7 (1/15)	7.7 (1/13)	0.99

*Abbreviations*: *n* sample size, *IOP* intraocular pressure as median (IQR), *BCVA* best-corrected visual acuity, *POAG* primary open angle glaucoma, *PXG* pseudoexfoliation glaucoma, *PG* pigmentary glaucoma, *Compounds* number of topical glaucoma medication, *IOP* intraocular pressure

### Surgical success

**Fig. 2:**
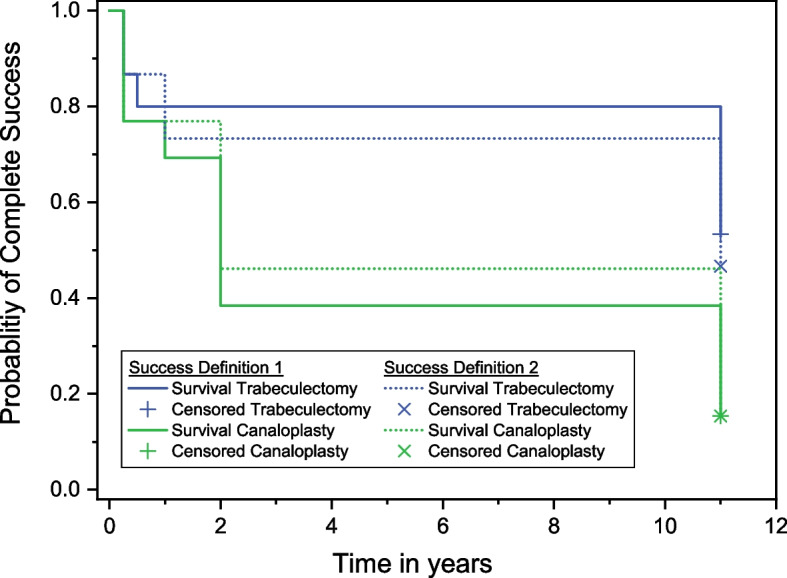
Kaplan–Meier survival analysis for complete success. Kaplan–Meier survival curves comparing the probability of complete success (without glaucoma medication) between trabeculectomy and canaloplasty. Solid lines represent survival curves under Definition 1 (IOP ≤ 18 mmHg), while dotted lines correspond to Definition 2 (IOP ≤ 21 mmHg AND ≥ 20% IOP reduction). For Definition 1, the curves differed significantly between both groups (*p* = 0.03). For Definition 2, no significance was reached (*p* = 0.09). *P*-values were calculated using log-rank test. Abbreviations: IOP = intraocular pressure

**Fig. 3:**
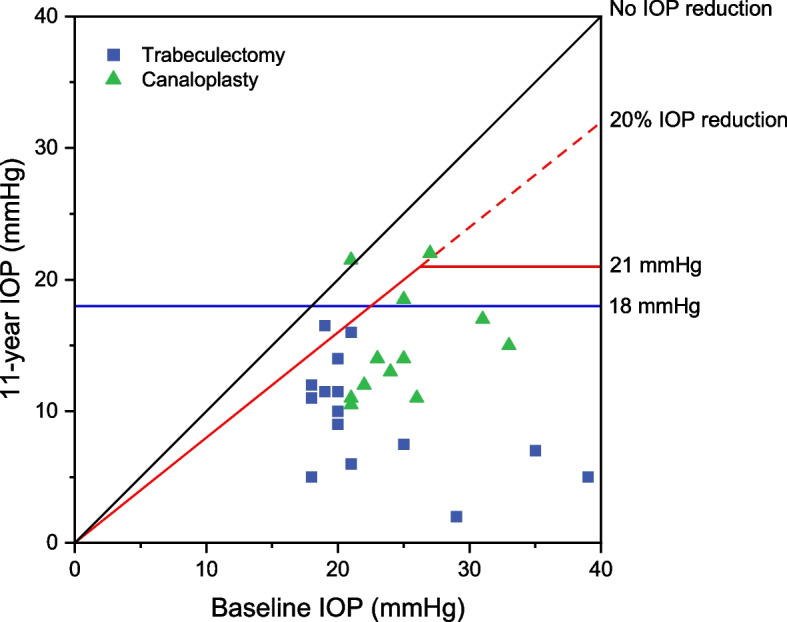
Scatter plot of intraocular pressure outcomes from baseline to 11-years follow-up. Scatter plot of preoperative intraocular pressure (IOP) (X-axis) and long-term follow-up (Y-axis) with or without glaucoma medication (qualified success). The squares and triangles represent single eyes. Eyes below the line of 18 mmHg were considered successful according to Definition 1 (blue line) while eyes below the 20%-reference line AND the horizontal 21 mmHg line met the success criteria for Definition 2 (red lines). Abbreviations: IOP = intraocular pressure, 11-year IOP = IOP at long-term follow-up (mean 11.5 ± 1.6 years), Baseline-IOP = preoperative IOP

At long-term follow-up, complete success according to Definition 1 (IOP ≤ 18 mmHg) was achieved in TE by 53.3% (*n *= 8) and in CP by 15.4% (*n* = 2). For Definition 2 (IOP ≤ 21 mmHg with ≥ 20% IOP reduction), complete success was met in TE by 46.7% (*n* = 6) and in CP by 15.4% (*n* = 2). In TE, the 95% confidence interval was 0.25–0.82 for Definition 1 and 0.18–0.75 for Definition 2, respectively. In CP, the 95% confidence interval ranged from −0.07 to 0.38 for both Definition 1 and Definition 2. Figure 2 illustrates the Kaplan–Meier survival analysis of the long-term probability for complete success in both groups. Both survival curves differed significantly at 11 years for Definition 1 (*p* = 0.03), whereas no difference was observed for Definition 2 (*p* = 0.09). For qualified success, Definition 1 was achieved in TE by 73.3% (*n* = 11) and in CP by 69.2% (*n* = 9). Under Definition 2, qualified success was achieved in TE by 66.7% (*n* = 10) and in CP by 76.9% (*n* = 10). No significant difference in the rate of qualified success was observed for either definition (*p* = 1.0 for Definition 1; *p* = 0.69 for Definition 2). All success rates are depicted in Supporting Table 1. A scatter plot illustrating IOP changes from baseline to the 11-year follow-up is presented in Fig. 3.

### IOP and compounds

Table 2 compares the IOP readings and compounds between both groups. Additionally, it includes data from the original TVC 2015 cohort for better comparison. In the long-term cohort, baseline IOP and IOP at 24 months were comparable between groups, whereas IOP at 12 months and 11 years showed a statistically significant difference. The number of compounds in the long-term cohort remained comparable across all time points. The absolute reduction in compounds at 11 years was 2.13 ± 1.6 in TE and 0.31 ± 1.7 in CP. Compared to baseline, this reduction was only significant in TE (*p* < 0.01) but not in CP (*p* = 0.47). The use of at least one compound was observed in 33% (5/15) of TE patients and 77% (10/13) of CP patients, a difference that was statistically significant (*p* = 0.03).

**Table 2:** Long-term results of intraocular pressure and compounds

	**Time Point**	**Trabeculectomy**			**Canaloplasty**			***p*-value cross-sectional**	
		**n**	**IOP**	**Compounds**	**n**	**IOP**	**Compounds**	**IOP**	**Compounds**
**TVC 2015 original cohort**	**Baseline**	32	20.0 (19.0–25)	3.3 ± 1.0	30	22.0 (20.0–26.25)	2.6 ± 1.8	0.06	0.09
	**12 months**	31	11.0 (8.0–12.0)	0.3 ± 0.7	23	13.0 (12.0–16.0)	0.8 ± 1.2	< 0.01	0.13
	**24 months**	31	11.0 (9.0–14.0)	0.4 ± 0.8	23	14.0 (11.0–18.0)	0.9 ± 1.1	< 0.01	0.04
**TVC 2025 long-term follow-up cohort**	**Baseline**	15	20.0 (16.0–21.0)	3.1 ± 0.8	13	23.0 (21.5–28.0)	2.2 ± 1.5	0.23	0.20
	**12 months**	15	10.0 (9.0–12.0)	0.5 ± 0.9	13	12.0 (10.0–16.0)	0.5 ± 1.2	0.04	0.93
	**24 months**	15	11.0 (9.0–13.0)	0.3 ± 0.8	13	12.0 (10.0–17.0)	0.7 ± 0.8	0.12	0.16
	**11 years**	15	10.0 (6.0–12.0)	1.0 ± 1.4	13	14.0 (11.5–17.75)	1.9 ± 1.5	< 0.01	0.11

*Abbreviations*: *n* sample size, *TVC* Trabeculectomy vs Canaloplasty, *Baseline* preoperative values, *Compounds* topical glaucoma medication as mean ± SD, *IOP* intraocular pressure as median (IQR), *cross-sectional* between both groups

**Fig. 4:**
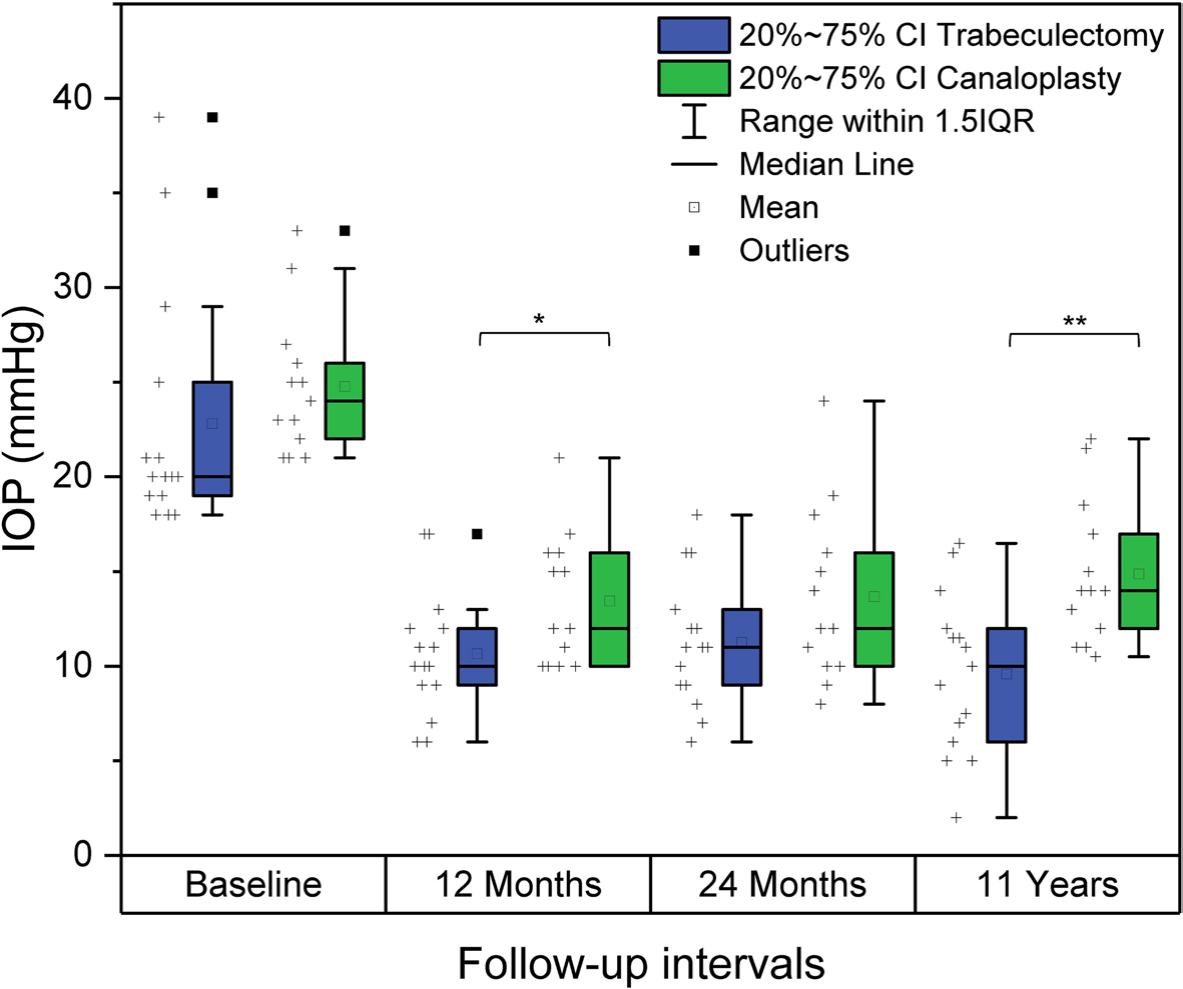
Box plots of intraocular pressure for all time points. IOP outcomes of both groups as box plots. The “ + ” symbols next to the boxes represent individual data points. At baseline and 24 months, IOP values did not differ significantly, while at 12 months and 11 years the readings differed significantly between both groups. Abbreviations: IOP = intraocular pressure, Baseline = preoperative IOP, * = significant at *p *≤ 0.05, ** = significant at* p* ≤ 0.01

IOP readings for the different time points are shown as box plots in Fig. 4. In a longitudinal comparison to the baseline IOP, long-term IOP was significantly lower in both groups (*p* < 0.01 for both). Mean IOP reduction from baseline to long-term follow-up was 11.6 ± 10.1 mmHg in TE and 9.4 ± 5.2 mmHg in CP. Compared to 12 months, IOP at 11 years did not differ significantly in both groups (TE *p* = 0.19 and CP *p* = 0.24). Similarly, compared to 24 months, the 11-year IOP was not significantly different in both TE (*p* = 0.06) and CP (*p* = 0.44). For this analysis, the number of compounds was not considered.

### Visual acuity, visual field, and SD-OCT

Best corrected visual acuity (BCVA) was reduced in both groups. At baseline, mean BCVA was 0.1 ± 0.2 logMAR in TE and 0.2 ± 0.3 logMAR in CP. Both values were comparable (*p* = 0.44). At long-term follow-up, mean BCVA in TE was 0.34 ± 0.3 logMAR and 0.43 ± 0.4 logMAR in CP (*p* = 0.52).

Mean defect (MD) at baseline was 10.43 ± 7.43 dB in TE and 4.57 ± 5.00 dB in CP. At long-term follow-up, mean defect was 14.28 ± 9.40 dB in TE and 7.29 ± 4.20 dB in CP. For each time point, the mean defect differed significantly between the two groups (*p *< 0.05 for both).

At 11 years, mean RNFL-thickness (G-value) assessed by SD-OCT was 62.4 ± 19.1 µm in TE and 66.1 ± 24.3 µm in CP.

### Surgical interventions and long-term complications

Only interventions and complications that occurred after the primary endpoint of the TVC study group were considered as long-term events. All pressure-lowering interventions were considered as revision surgeries. In TE, 20.0% (3/15) underwent subsequent glaucoma surgery. In CP, the proportion was 23.1% (3/13). For those patients who received further surgery, the time to intervention was 63 ± 33.5 months in TE and 96 ± 43.6 months in CP. Hypotony maculopathy, defined as IOP ≤ 5 mmHg with macular folds on OCT, was the only long-term complication, occurring in 13.3% (2/15) of TE patients (Supporting Table 2).

## Discussion

This prospective long-term follow-up evaluates the outcomes of trabeculectomy and canaloplasty in patients from the original TVC trial cohort [2].

At 11 years, TE was superior regarding IOP and cumulative complete success for Definition 1 (IOP ≤ 18 mmHg). However, there was no difference in complete success for Definition 2 (IOP ≤ 21 mmHg AND 20% IOP reduction). Qualified success did not differ between TE and CP patients for either definition.

In the original trial, trabeculectomy demonstrated superior complete success at 24 months for both definitions [2]. However, the reduced sample size in this subsequent study renders it more difficult to demonstrate substantial differences at a *p*-value of 0.05. Despite this limitation, the confidence intervals suggest a trend towards higher complete success rates for Definition 2 in TE.

Even after 11 years, both procedures effectively lower IOP but TE demonstrates greater efficacy in reducing medication use and achieving medication-free outcomes. Yet, this comes at the cost of an increased risk of complications, with hypotony maculopathy observed exclusively in TE. For patients with moderately low target IOPs who can tolerate some glaucoma medication use, CP remains a reliable and safer alternative.

We were able to follow up approximately 50% of the original cohort. This highlights the challenges of collecting long-term outcome data. These challenges include patient relocation, inability to visit due to declining health, or mortality. To the best of our knowledge, our study reports the longest follow-up of a randomized comparison between trabeculectomy and canaloplasty. Despite these challenges, some studies have successfully conducted long-term follow-up of patients who have undergone trabeculectomy and canaloplasty.

To our knowledge, only one study has compared modified ab externo canaloplasty with trabeculectomy over a follow-up of more than four years [7]. At a median follow-up of 4.6 years, canaloplasty patients had a mean IOP of 13.1 ± 3.3 mmHg and used 0.8 ± 1.0 compounds, while trabeculectomy achieved a lower mean IOP of 11.3 ± 3.6 mmHg and 0.1 ± 0.3 drops. Trabeculectomy also showed superior surgical success, with 66.4% complete success (IOP 5–18 mmHg without medication and revision surgery) compared to 24.3% for canaloplasty. However, the use of Mitomycin C in the modified canaloplasty limits direct comparability with conventional techniques.

Landers et al. reported that 57% of trabeculectomy patients maintained complete success (IOP ≤ 21 mmHg without medication) after 20 years, with a qualified success rate of 88% [8]. Trabeculectomy also demonstrated durability regarding the need for additional glaucoma surgeries, with Chen et al. showing a 15-year probability of 67% for further surgery. However, their definition of success differed and 77.5% (31/40) of the patients resumed glaucoma medication after 41 months [9]. To date, only one study has conducted a follow-up on trabeculectomy patients within a German cohort comparable to ours. Wagner et al. found a general success rate (IOP ≤ 18 mmHg and 20% reduction) of 75% after 5 years, with 67% complete success. At the last follow-up, mean IOP was 12.1 ± 4.3 mmHg, with 0.07 ± 0.26 compounds [10]. These results are comparable to our findings.

Long-term data for canaloplasty, particularly ab externo canaloplasty, is less extensive.

Tognetto et al. compared mid-term outcomes of ab externo canaloplasty and phacocanaloplasty in 30 eyes. At 42 months, complete success without glaucoma medication and IOP of ≤ 18 mmHg was achieved in 20% of the patients, while qualified success was reached in all patients. Mean IOP was 14.3 ± 3.0 mmHg, with an average medication count of 1.7 ± 1.7 drops [11].

Three-year results of a prospective multicenter trial by Lewis et al. show that canaloplasty and phacocanaloplasty are both efficient and safe in treating open-angle glaucoma. Mean IOP three years postoperatively was 15.2 ± 3.5 mmHg, achieved on 0.8 ± 0.9 drops [12].

In 2014, Brusini retrospectively analyzed the three-year outcome of ab externo canaloplasty in 29 patients. At 36 months, mean IOP was 17.3 ± 3.9 mmHg, and the average number of compounds was 1.3 ± 1.5. Qualified and complete success rates, defined similarly to our methods, were 58.6% and 31.0%, respectively [13].

Ennerst et al. reported a longer follow-up of 109 months in a retrospective analysis of 48 eyes, comparing canaloplasty and phacocanaloplasty with similar efficacy in IOP reduction. At 10 years, the mean IOP was 14.8 ± 2.0 mmHg, with an average medication use of 0.5 ± 0.9 compounds [14].

The IOP findings regarding the long-term efficacy of canaloplasty align with our results on long-term efficacy of canaloplasty. Similarly, the number of medications appears to be generally comparable with exceptions noted in the findings of Ennerst et al. and Stingl et al. [7, 14].

Beyond long-term IOP reduction and medication use, the occurrence of late complications is a crucial parameter. Non-penetrating procedures like canaloplasty are generally safer than trabeculectomy [15]. Transient hyphema is a common complication of canaloplasty, occurring in 21–40%, while hypotony rates are low (0.6–20%) [7, 16]. In contrast, trabeculectomy has higher complication rates (7–40%), including hypotony maculopathy (4–7%) and bleb-related complications (1.6–23%) [16–18]. In our cohort, hypotony maculopathy was the only long-term complication occurring in 15.4% (2/13) after trabeculectomy. These findings highlight possible detrimental consequences of excessively low IOP, reinforcing the relevance of incorporating both upper and lower IOP thresholds when defining surgical success. No bleb-related complications were observed.

Collecting long-term data remains a significant challenge, as seen in the small sample sizes of many studies and our own cohort. However, such data is vital for evaluating the long-term efficacy of glaucoma procedures. We emphasize the need for prospective long-term studies and extensive registries to generate reliable data and support individualized treatment approaches.

While we achieved sufficient power (0.8) for CP by including 13 of 23 former patients from the 24-month primary endpoint, the comparison for TE lacked power, with only 15 of 31 patients included. Achieving a power of 0.8 required at least 50% of former patients, highlighting a key study limitation. Nevertheless, the relatively small overall sample size may limit the generalizability of the findings and should be taken into consideration when interpreting the results. Another limitation is reliance on community-based practitioner data, which may introduce bias despite consistent use of Goldmann applanation tonometry. The significant difference in the visual field mean defect between TE and CP suggests more severe glaucoma in the TE group. Although the original cohort was randomized, it is unclear if this discrepancy existed at baseline. *The similar rate of visual field progression suggests that both surgical procedures provided comparable long-term IOP control, which likely contributed to a similar rate of structural and functional decline. Some continued deterioration is expected given the chronic nature of glaucoma and the extended follow-up period. Additionally, the advanced age of participants at follow-up may affect visual field reliability due to factors such as fatigue, reduced concentration, or comorbidities. Nevertheless, the parallel trends in functional and structural parameters support the internal consistency of our findings.*

Despite its limitations, the study’s long follow-up is a major strength, offering valuable insights into long-term outcomes of both procedures. The consistent mid-term results reinforce the reliability of the findings and highlight the importance of long-term data in clinical decision-making. Our results support evidence-based surgical recommendations, demonstrating that both procedures remain widely used after 11 years, underscoring the challenge of new alternatives and trabeculectomy’s enduring relevance in glaucoma surgery.

## Conclusion

In conclusion, this study provides valuable long-term insights from a predefined cohort of trabeculectomy and canaloplasty patients, reevaluated over 11 years. It also emphasizes the difficulty and importance of collecting prospective data over such an extended period. Despite the challenges, our findings highlight that trabeculectomy remains more effective for achieving lower intraocular pressure and reducing medication use. However, canaloplasty proves to be a safe and effective alternative when some medication use is acceptable, and the target IOP is not extremely low. The enduring use of both procedures underscores their lasting relevance in glaucoma surgery and the critical role of long-term data in shaping evidence-based clinical decisions.

## Supplementary Information

Supplementary Material 1.

Supplementary Material 2.

## Data Availability

Data is available from the corresponding author on request.

## Abbreviations

TVC: Trabeculectomy versus Canaloplasty
IOP: Intraocular pressure
TE: Trabeculectomy
CP: Canaloplasty
BCVA: Best-corrected visual acuity
POAG: Primary open angle glaucoma
PXG: Pseudoexfoliation glaucoma
PG: Pigmentary glaucoma
