# Supplementary material for: Long-term outcomes of trabeculectomy versus canaloplasty in open-angle glaucoma – an 11-year follow-up of the TVC study cohort
Source: BMC Ophthalmol. 2025 Jun 16;25:340. doi: 10.1186/s12886-025-04183-9 (PMC12168381; doi:10.1186/s12886-025-04183-9)
Supplement: Supplementary file 1 — Supplementary Material 1. [file 12886_2025_4183_MOESM1_ESM.docx]

**Supporting Table 1 – Surgical success of trabeculectomy and canaloplasty patients**

| **Criteria** | | | **Trabeculectomy  (n = 15)** | | **Canaloplasty**  **(n = 13)** | | **p-value** |
| --- | --- | --- | --- | --- | --- | --- | --- |
|  |  |  | **n/N** | **%** | **n/N** | **%** |  |
| **Complete success** | Definition 1 | 12 months  24 months  11 years | 11/15  12/15  8/15 | (73.3) (80.0) (53.3) | 8/13  5/13  2/13 | (61.5)  (38.5)  (15.4) | 0.70  0.05  0.06 |
|  | Definition 2 | 12 months  24 months  11 years | 10/15  9/15  7/15 | (66.7)  (60.0)  (46.7) | 7/13  5/13  2/13 | (53.8)  (38.5)  (15.4) | 0.38  0.45  0.11 |
| **Qualified success** | Definition 1 | 12 months  24 months  11 years | 15/15  15/15  11/15 | (100.0)  (100.0)  (73.3) | 11/13  11/13  9/13 | (84.6)  (84.6)  (69.2) | 0.21  0.21  1.00 |
|  | Definition 2 | 12 months  24 months  11 years | 13/15  12/15  10/15 | (86.7)  (80.0)  (66.7) | 11/13  11/13  10/13 | (84.6)  (84.6)  (76.9) | 1.00  1.00  0.69 |

P-values were calculated using Fisher's exact test.

Abbreviations: n/N = number of cases out of the total sample, Complete = success without medication use, IOP = intraocular pressure, Qualified = success regardless of medication use, Definition 1 = IOP ≤ 18 mmHg, Definition 2 = IOP ≤ 21 mmHg AND ≥ 20% IOP reduction.
