# Supplementary material for: Long-term outcomes of trabeculectomy versus canaloplasty in open-angle glaucoma – an 11-year follow-up of the TVC study cohort
Source: BMC Ophthalmol. 2025 Jun 16;25:340. doi: 10.1186/s12886-025-04183-9 (PMC12168381; doi:10.1186/s12886-025-04183-9)
Supplement: Supplementary file 2 — Supplementary Material 2. [file 12886_2025_4183_MOESM2_ESM.docx]

**Supporting Table 1 – Complications and interventions at long-term follow-up**

|  | **Trabeculectomy (n = 15)** | | **Canaloplasty (n = 13)** | |
| --- | --- | --- | --- | --- |
| **Complications** | **Proportion** | | **Proportion** | |
| Hypotony Maculopathy | 13.3% (2/15) | | 0 % | |
|  |  |  |  |  |
| **Interventions** | **Proportion** | **Time to event in months** | **Proportion** | **Time to event in months** |
| Second glaucoma surgery | 20% (3/15) | 63 ± 33.5 | 23.1% (3/13) | 96 ± 43.6 |
| Bleb revision | 2/3 | 44 ± 8.5 | - | - |
| Ab interno trabeculectomy (Trabectome) | 1/3 | 101 | - | - |
| CyPass® Implantation | - | - | 1/3 | 59 |
| Trabeculectomy | - | - | 1/3 | 85 |
| PRESERFLO® Implantation | - | - | 1/3 | 144 |

Hypotony maculopathy was defined as IOP of ≤ 5 mmHg and the presence of macular folds on OCT.

Abbreviations: n = sample size, IOP = intraocular pressure
